# Predictive Accuracy of a Clinical Model for Carriage of Pathogenic/Likely Pathogenic Variants in Patients with Dementia and a Positive Family History at PUMCH

**DOI:** 10.3390/biomedicines13051235

**Published:** 2025-05-19

**Authors:** Jialu Bao, Yuyue Qiu, Tianyi Wang, Li Shang, Shanshan Chu, Wei Jin, Wenjun Wang, Yuhan Jiang, Bo Li, Yixuan Huang, Bo Hou, Longze Sha, Yunfan You, Yuanheng Li, Meiqi Wu, Yutong Zou, Yifei Wang, Li Huo, Ling Qiu, Qi Xu, Feng Feng, Chenhui Mao, Liling Dong, Jing Gao

**Affiliations:** 1 State Key Laboratory of Complex Severe and Rare Diseases, Department of Neurology, Peking Union Medical College Hospital, Chinese Academy of Medical Sciences and Peking Union Medical College, Peking Union Medical College Hospital Translational Medical Center, Beijing 100730, China; 1710122524@pku.edu.cn (J.B.); qiuyuyue2022@163.com (Y.Q.); wtyi@pku.edu.cn (T.W.); shangl13@126.com (L.S.); chushanshan2025@163.com (S.C.); jinwei44@sina.cn (W.J.); iceplusdeer@163.com (W.W.); jiangyuhan@pumch.cn (Y.J.); l-b17@mails.tsinghua.edu.cn (B.L.); huangxiuxiu70@foxmail.com (Y.H.); ggyunfan@gmail.com (Y.Y.); s2024001032@student.pumc.edu.cn (Y.L.); maochenhui@pumch.cn (C.M.); 2 Department of Radiology, Peking Union Medical College Hospital, Chinese Academy of Medical Sciences and Peking Union Medical College, Beijing 100730, China; houbo97@pumch.cn (B.H.); ffeng@pumch.cn (F.F.); 3 State Key Laboratory of Medical Molecular Biology, Institute of Basic Medical Sciences, Chinese Academy of Medical Sciences, School of Basic Medicine Peking Union Medical College, Beijing 100730, China; shalz_pumc@163.com (L.S.); xuqi@pumc.edu.cn (Q.X.); 4 State Key Laboratory of Complex Severe and Rare Diseases, Department of Nuclear Medicine, Center for Rare Diseases Research Beijing Key Laboratory of Molecular Targeted Diagnosis and Therapy in Nuclear Medicine, Peking Union Medical College Hospital, Chinese Academy of Medical Science and Peking Union Medical College, Beijing 100730, China; wumqpumch@126.com (M.W.); huoli@pumch.cn (L.H.); 5 Department of Laboratory Medicine, Peking Union Medical College Hospital, Chinese Academy of Medical Science and Peking Union Medical College, Beijing 100730, China; ytongz@126.com (Y.Z.); yifeiwang5911@163.com (Y.W.); qiul@pumch.cn (L.Q.)

**Keywords:** dementia, family history, age at onset, whole exome sequencing, *APOE* ε, clinical prediction model, Pathogenic/Likely Pathogenic Variants

## Abstract

**Background and Objectives:** Identifying carriers of Pathogenic/Likely Pathogenic Variants in patients with dementia is crucial for risk stratification, particularly in individuals with a family history. This study developed and validated a clinical prediction model using whole-exome sequencing-confirmed cohorts. **Methods:** A total of 601 Chinese patients with dementia and a family history were enrolled at Peking Union Medical College Hospital, with 476 in a retrospective derivation cohort and 125 in a temporal validation cohort. Predictive factors included age at onset, *APOE* ε4 status, and family history characteristics. Model performance was assessed using discrimination and calibration metrics. **Results:** In the derivation cohort (median age at onset 66 years), 10.3% carried Pathogenic/Likely Pathogenic Variants. Among patients with dementia, those with age at onset < 55 years (OR 2.56, *p* = 0.0098), more than two affected relatives (OR 3.32, *p* = 0.0039), parental disease history (OR 4.72, *p* = 0.015), and early-onset cases in the family (OR 2.61, *p* = 0.0096) were positively associated with Pathogenic/Likely Pathogenic Variant carriage, whereas *APOE* ε4 carriage was inversely associated (OR 0.36, *p* = 0.0041). The model achieved an area under the curve of 0.776 (95% CI, 0.701–0.853) in the derivation cohort and 0.781 (95% CI, 0.647–0.914) in the validation cohort (median age at onset 58 years), with adequate calibration. **Conclusions:** This model demonstrated strong predictive performance for Pathogenic/Likely Pathogenic Variant carriage, supporting its clinical utility in guiding genetic testing. Further research is needed to refine the model.

## 1. Introduction

Dementia prevalence is rising among the aging population [1]. Diagnostic approaches for both disease identification and staging now incorporate biomarkers and genetic testing [2], but the high costs and lengthy turnaround times associated with techniques like whole-exome sequencing (WES) constrain their routine clinical application [3,4,5]. Consequently, accurately identifying carriers of Pathogenic/Likely Pathogenic Variants (P/LP Variants), particularly among patients with dementia and a positive family history, could streamline genetic testing, lower costs, and improve clinical risk stratification.

Traditional family history scoring systems (e.g., the Goldman and Penn scores) provide useful frameworks but exhibit key limitations. For example, the Goldman scoring system categorizes patients with a positive family history into four levels: autosomal dominant inheritance, familial recurrence, early onset, and the presence of dementia in the family [6]. However, they often fail to differentiate risk when multiple affected relatives are present alongside early-onset cases and may be hindered by incomplete three-generation data. In contrast, the Penn score, which focuses on describing both disease types and intergenerational family history relationships, was developed as an improved standard specifically for frontotemporal dementia (FTD); however, its applicability may be limited to that disease spectrum [7]. Furthermore, these scoring systems do not fully leverage a comprehensive range of clinical features, such as age at onset, family history, disease type, gender, cognitive function, and *APOE* genotype, to enhance prediction accuracy [8,9,10]. Integrating these diverse factors holds promise for improving the identification of carriers of P/LP Variants.

Early identification of P/LP Variant carriers offers considerable clinical advantages. For example, patients with dementia carrying P/LP Variants in genes such as *APP*, *PSEN1*, and *PSEN2* exhibit distinct prognoses compared to those with sporadic Alzheimer’s disease [11]. Recent expert consensus emphasizes comprehensive disease management across the entire clinical course, with particular attention to preclinical diagnosis. Within the NSD-ISS framework [12], an early gene-based risk staging (pre-NSD0 stage) is proposed, and the HD-ISS framework similarly highlights the significance of focusing on patients at stage 0 (with ≥40 CAG repeats) [13]. Individuals carrying highly penetrant P/LP Variants may receive a definitive molecular diagnosis even before symptom onset, thereby enabling timely intervention and tailored follow-up. Although our study targets already diagnosed patients with dementia, it is important to note that many dementia-related pathogenic genes follow an autosomal dominant inheritance pattern, meaning that first-degree relatives might share up to a 50% risk [14]. Such early detection can facilitate the application of preventive strategies, including enrollment in clinical trials and provision of psychological support for at-risk family members.

To address these limitations, we aimed to construct and validate a predictive model integrating accessible clinical variables (age at onset, family history, dementia subtypes, neuropsychological assessments) with genetic risk indicators (*APOE* ε4 status) to estimate the likelihood of P/LP Variant carriage in patients with dementia and a positive family history. Our approach circumvents the Goldman score’s dependency on complete pedigrees by focusing on first-degree relatives’ disease patterns, while extending the Penn score’s scope through cross-subtype applicability. By standardizing risk stratification using routinely collected data, this study seeks to bridge a critical gap in guiding genetic testing decisions.

## 2. Methods

### 2.1. Participants

**Figure 1:**
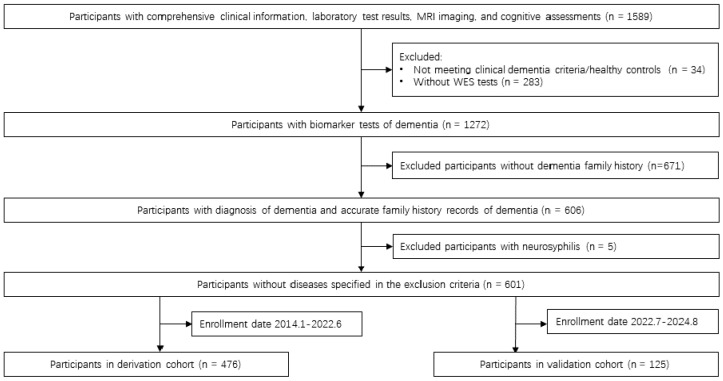
Derivation and validation cohorts used to develop the prediction model for Pathogenic/Likely Pathogenic Variant carriage among patients with dementia and a family history. Flowchart of participant enrollment and cohort selection. Participants from the PUMCH dementia biomarker cohort underwent clinical assessments, laboratory testing, MRI imaging, and cognitive evaluations. Exclusion criteria included lack of dementia diagnosis, absence of whole-exome sequencing (WES), no family history of dementia, and presence of neurosyphilis. The final eligible cohort (*n* = 601) was divided into derivation (*n* = 476; January 2014–June 2022) and temporal validation (*n* = 125; July 2022–August 2024) cohorts based on enrollment dates.

The Peking Union Medical College Hospital (PUMCH) dementia biomarker cohort is a 10-year prospective study initiated in January 2014, recruiting patients from the PUMCH dementia clinic across 33 provinces in China. A total of 1589 participants with comprehensive clinical data, laboratory tests, MRI imaging, and cognitive assessments were initially enrolled. After excluding 34 individuals who did not meet clinical dementia criteria or were healthy controls and 283 without WES tests, 1272 participants underwent dementia biomarker testing. Subsequently, 671 individuals lacking a family history of dementia were excluded, leaving 606 participants with confirmed dementia diagnoses and documented family history. Five cases with neurosyphilis were further excluded, resulting in 601 eligible participants. Based on enrollment periods, these were divided into a derivation cohort (*n* = 476; enrolled January 2014–June 2022) and a validation cohort (*n* = 125; enrolled July 2022–August 2024) (Figure 1).

### 2.2. Eligibility Criteria

To participate, individuals were required to have comprehensive clinical history records and family history of dementia. Baseline data collection included blood tests, systemic cognition assessment, MRI imaging, whole-exome sequencing and dynamic mutation sequencing. All participants were clinically diagnosed with dementia.

### 2.3. Exclusion Criteria

Participants were excluded if they had a history of severe traumatic brain injury, carbon monoxide poisoning, occupational exposure to toxic substances, malignant tumors of the nervous system, thyroid dysfunction (hyperthyroidism or hypothyroidism), chronic infectious diseases (including hepatitis B, syphilis, and HIV), or no family history of dementia.

### 2.4. Diagnosis Criteria

Diagnosis of dementia followed the NIA-AA 2011 guidelines [15], requiring significant cognitive decline in at least two domains affecting daily activities, with other causes excluded. Standardized tools used for assessment included the Mini-Mental State Examination (MMSE), the Montreal Cognitive Assessment (MoCA), and the Activities of Daily Living (ADL) scale, and the PUMCH Comprehensive Dementia Scale covering memory, language, visuospatial abilities, executive function, logical reasoning, and calculation. All assessments were conducted by certified neuropsychological evaluators who underwent standardized training. Diagnoses were reviewed and confirmed by experienced neurologists. Alzheimer’s disease diagnosis followed the NIA-AA Criteria for Diagnosis and Staging of Alzheimer’s Disease with AD-related biomarkers [2]. Frontotemporal dementia diagnosis was based on the Rascovsky [16] and Gorno–Tempini [17] criteria. Prion disease diagnosis was based on the Hermann guidelines [18] with RT-QuIC by the China CDC. Vascular dementia diagnosis was based on the NINDS-AIREN criteria [19].

### 2.5. Clinical and Laboratory Evaluations

Participants underwent routine blood tests, including hematology, biochemistry, metabolic panels, and infectious disease screening. *APOE* ε4 genotyping, thyroid function tests, ESR, CRP, and electrolyte levels were also assessed. MRI imaging, including 3D T1-weighted, T2-weighted, fluid-attenuated inversion recovery (FLAIR), diffusion-weighted imaging (DWI), apparent diffusion coefficient (ADC), and susceptibility-weighted imaging (SWI)/susceptibility-weighted angiography (SWAN) sequences, was performed for all participants. All participants completed MMSE, MoCA, and ADL tests. MMSE used education-adjusted cutoffs (dementia if ≤19 for ≤6 years education; ≤23 for >6 years). MoCA scores were adjusted (+2 points for ≤6 years; +1 for 7–12 years). These adjusted scores were used to define dementia and included as predictors in the regression model.

### 2.6. Genetic and Biomarker Analyses

Genomic DNA was extracted from peripheral blood leukocytes. WES libraries were prepared and sequenced on both the Illumina HiSeq X Ten (Illumina, San Diego, CA, USA) and the MGI DNBSEQ platforms (MGI Tech, Shenzhen, China). Both platforms generated 150 bp paired-end reads, targeting an average on-target coverage depth of ≥100×, with ≥95% of exonic regions covered at ≥20×. Variants were annotated using ANNOVAR v2019Oct24 against the hg38_refGene table (RefSeq transcripts updated at UCSC on 17 August 2020; downloaded 19 October 2021). Allele frequencies and clinical classifications were obtained from ClinVar (build 2024-12; downloaded December 2024) and gnomAD r3 (downloaded January 2025). HGVS nomenclature was validated with Mutalyzer v2.0.35. Rare (MAF < 0.5%) nonsynonymous, splice-site, and loss-of-function variants were classified per ACMG 2015 guidelines [20], using in silico predictions to prioritize damaging changes. Only individuals with autosomal dominant inheritance carrying P/LP Variants, and individuals with autosomal recessive inheritance carrying one P/LP variant and at least one VUS, were carried forward into downstream analyses. Dynamic repeat expansions in *C9orf72*, *NOTCH2NLC*, *HTT*, and *FMR1* were screened by repeat-primed PCR and capillary electrophoresis, with established pathogenic repeat thresholds applied for each gene [21]. In the Alzheimer’s disease cohort, all P/LP variant carriers identified by WES underwent Sanger sequencing, except for patients who met the NIA-AA 2011 criteria for probable AD and carried *APP*, *PSEN1*, or *PSEN2* variants.

Biomarker evaluation included AD-related biomarker tests, such as cerebrospinal fluid (CSF) measurements of Aβ40, Aβ42, tTau, and pTau [22,23], or amyloid imaging using Pittsburgh B compound/[18F]-Florbetazine [24] and Tau-PET imaging (MK6240) [25].

The study was approved by the Coordinating Ethics Committee of Peking Union Medical College Hospital. All participants provided written informed consent during the screening and baseline visits.

### 2.7. Study Design

We collected detailed clinical and family history data from participants with dementia and a positive family history in the PUMCH cohort. Using reported proportions of P/LP in dementia cohorts, we calculated the minimum sample size for model construction. Variables related to P/LP factors were identified based on family history classifications, clinical characteristics, *APOE* ε4 status, gender, cognitive function, and disease classification. Univariate associations were evaluated by Fisher’s exact test for categorical predictors and by global Wald tests for restricted cubic splines in continuous predictors. Variables with *p* < 0.05 or clear clinical relevance were then included in a multivariable logistic regression model. To evaluate the model’s performance, we conducted validity tests, including the calculation of the area under the receiver operating characteristic curve (AUC) and calibration assessments using the Hosmer–Lemeshow test and calibration plot.

### 2.8. Sample Size Calculation

Based on previous literature, the proportion of P/LP mutations in dementia cohorts is approximately 1–10% [26,27,28]. To ensure an adequate sample size, we used an anticipated outcome proportion of 10% for calculations [29].

We set a 5% margin of error to balance precision with practical feasibility and used a 10% anticipated P/LP detection rate, reflecting upper-range literature values, to ensure sufficient power. However, P/LP rates vary by ethnicity, onset criteria, and sequencing methods, so our Chinese WES-plus-dynamic mutation cohort may differ.

#### 2.8.1. Estimation of Overall Outcome Proportion

$$n={\left(\frac{1.96}{\delta }\right)}^{2}\times \hat{\varphi }\times \left(1-\hat{\varphi }\right)$$

where

Margin of Error: (δ) = 0.05

Anticipated Outcome Proportion: ($\hat{\varphi }$) = 0.1:

$$n={\left(\frac{1.96}{0.05}\right)}^{2}\times 0.1\times \left(1-0.1\right)\;=\;138.3$$

Rounded up, 139 participants are required to ensure the precision of the overall outcome proportion.

#### 2.8.2. Prediction Model with Small Mean Absolute Error (MAPE)

The formula for calculating the sample size for a small mean absolute error (MAPE) is:

$$\ln\left(MAPE\right)=-0.508-0.544\ln\left(n\right)+0.259\ln\left(\hat{\varphi }\right)+0.504\ln\left(P\right)$$

Solving for *n* yields 145 participants needed for a small mean absolute error in predicted probabilities.

#### 2.8.3. Shrinkage of Predictor Effects

The sample size for shrinkage of predictor effects is calculated using the formula:

$$n=\frac{P}{\left(S-1\right)\ln\left(1-\frac{R_{cs}^{2}}{S}\right)}$$

where *S* is:

$$S=R_{cs}^{2}/\left(R_{cs}^{2}+\delta \times max\left(R_{cs}^{2}\right)\right)\;=\;0.8857$$

For this study, *S* = 0.8857. Therefore:

$$n=P/\left[\left(S-1\right)\times ln\left(1-R_{cs}^{2}/S\right)\right]\;=\;167$$

Thus, 167 participants are required to ensure minimal shrinkage of predictor effects.

### 2.9. Explanatory Variables

Descriptive variables were extracted from the PUMCH electronic medical record system, including age at onset, gender, family history, *APOE* ε4 carrier status genotype, diagnosis, and neuropsychological testing. Following univariate analysis, literature review, and comparisons of various multivariate models, the final regression incorporated five key variables: age at onset, family history (including the number of affected relatives, parental disease status, and the presence of early-onset cases), and *APOE* ε4 carrier status.

“Age of onset” (AAO) was calculated as the difference between the time of the first visit and the disease onset reported in the patient’s chief complaint. “*APOE* ε4 carrier status” includes both *APOE* ε4/- and *APOE* ε4/ε4. Family history includes only first-degree to third-degree relatives. The “number of affected relatives” (RelNum) refers to the number of relatives within three generations who have been diagnosed with dementia. “Parental disease status” includes cases where one or both parents are affected by dementia. “Presence of early-onset cases” (EarlyFH) refers to any relative within three generations who developed dementia before the age of 65. Diagnosis was based on the 2011 NIA-AA guidelines, classifying participants into Alzheimer’s disease (AD), frontotemporal dementia (FTD), vascular dementia (VaD), or other dementia-related diseases. Neuropsychological assessments included the MMSE, MoCA, and the rate of ADL progression, with each measure calculated as the difference between the final and initial evaluation scores, divided by the time interval between the two assessments.

### 2.10. Outcome Measures

The primary outcome was carriage of P/LP Variants as defined in Section 2.6. Model performance was evaluated by discrimination (area under the ROC curve with 95% CI) and calibration (Hosmer–Lemeshow test and calibration plot). Clinical utility was assessed by decision curve analysis (DCA) using the R package rmda v1.6, calculating net benefit across threshold probabilities from 0 to 1 in 0.01 increments.

### 2.11. Statistical Analysis

Descriptive statistics are presented as median (interquartile range, IQR) for continuous variables and frequency for categorical variables. Between-group comparisons were performed using the Fisher’s exact test for categorical variables. Age, RelNum, MMSE, MoCA and ADL progression were fitted using restricted cubic splines with three knots at the 10th, 50th, and 90th percentiles, while other variables were categorized into binary variables based on the proportion of P/LP variant frequency.

In this study, only *APOE* genotype data were missing <3% (18/601). Because the proportion of missing data was low and could be considered missing at random, we used population mode imputation for these data. To assess the impact of imputation on our findings, we also conducted a complete-case analysis, which showed no significant difference in the results.

A multivariate regression analysis was performed using five variables to analyze the model parameters. *p* < 0.05 and a 95% two-sided confidence interval that does not include 1 were considered statistically significant. Based on sample size calculations, a minimum of 167 patients was required, and our derivation cohort included 476 patients, exceeding this threshold. To maximize model stability, we adhered to 9.5 events per predictor degree of freedom, therefore the sample size is sufficient to support the study conclusions. For model performance evaluation, we used the area under the receiver operating characteristic curve (AUC) as a comprehensive measure. Model stability was assessed using the Hosmer–Lemeshow test and calibration curve plotting. All data analysis and plots were performed using R statistical software, version 4.2.4.

### 2.12. Subgroup ROC and Calibration Analysis by Inheritance Mode

We additionally evaluated model performance separately in autosomal-dominant and autosomal-recessive carriers versus non-carriers. For each subgroup, we calculated the ROC curve and AUC (95% CI) using the pROC package in R, and assessed calibration by plotting observed versus predicted probabilities by decile and performing the Hosmer–Lemeshow test. All analyses were conducted in R version 4.2.4.

## 3. Results

A total of 601 patients with clinically diagnosed dementia were enrolled in this cohort, including 409 patients with AD, 45 with FTD, 63 with VaD, and 84 with other dementia subtypes (Supplementary Table S1). The initial 476 patients enrolled from January 2014 to June 2022 comprised the derivation cohort, and the subsequent 125 patients enrolled from July 2022 to August 2024 comprised the validation cohort (Table 1).

In 601 participants, WES identified 62 carriers (10.3%) of 24 distinct P/LP variant loci, including a first-degree relative trio (IDs 229, 248, 249). For validation, we performed Sanger sequencing on 17 P/LP carriers. The validation set comprised three APP-mutation CAA cases (Boston criteria), one CADASIL-like case, nine non-AD gene carriers (e.g., *VCP, PDGFRB*), and four additional randomly selected carriers. Two samples (IDs 493 and 447) failed PCR; all 15 successfully sequenced samples showed 100% concordance with WES. Full details of the 24 distinct P/LP variant loci and baseline clinical information for all 62 carriers are provided in Supplementary Table S8 (Excel file).

In the derivation cohort of 476 patients (median age at onset, 66 [IQR 58–73] years; 196 men [41.2%]), 40 (8.4%) carried *APOE* ε4/ε4, 164 (34.5%) carried *APOE* ε4/–, and 49 (10.3%) carried P/LP Variants. The median Goldman score was 3.5 (IQR 3.5–3.5), with median MMSE 18 (IQR 11–24), MoCA 16 (IQR 12–21), and ADL 34 (IQR 26–44). In the validation cohort of 125 patients (median age at onset, 58 [IQR 52–67] years; 52 men [41.6%]), 10 (8.0%) carried *APOE* ε4/ε4, 40 (32.0%) carried *APOE* ε4/–, and 13 (10.4%) carried P/LP Variants. Baseline characteristics differed slightly between the derivation and temporal validation cohorts due to sequential sampling.

**Figure 2:**
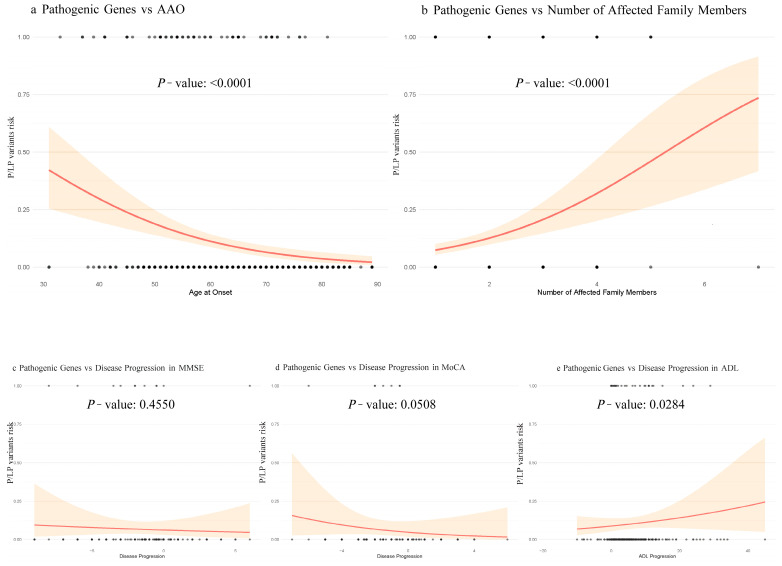
Correlation of AAO, RelNum, and cognition with P/LP Variant detection rate. The shaded area represents the 95% CI from the restricted cubic spline models. Panel (**a**) shows the relationship between P/LP Variant detection rate and AAO; Panel (**b**) shows the relationship between P/LP Variant detection rate and RelNum. Panels (**c**–**e**) display P/LP Variant detection rate versus progression rates in MMSE, MoCA, and ADL, respectively. Cognitive progression rates were calculated as (final score − baseline score)/years of follow-up.

We used univariate logistic regression and restricted cubic splines to evaluate associations of AAO, RelNum, and cognitive decline with P/LP Variant detection. P/LP Variant detection decreased progressively and then stabilized as AAO increased (nonlinear component *p* = 0.61). By contrast, higher RelNum was strongly associated with greater P/LP Variant detection (*p* < 0.001). Among cognitive measures, faster ADL deterioration correlated with higher detection rates (*p* = 0.028), whereas MMSE and MoCA showed no significant associations (*p* > 0.05) (Figure 2).

Based on previous literature and the characteristics of our cohort, we found that EarlyFH and inheritance patterns were significantly associated with P/LP Variant detection rate in patients with dementia and a family history. Due to challenges in applying autosomal dominant inheritance patterns in clinical practice and incomplete family history recall, we used parental disease status as a substitute, which also showed a very large difference (*p* = 0.017). Among patients with a family history of dementia, those with EarlyFH had a higher P/LP Variant detection rate than those without EarlyFH (24.59% vs. 6.68%, *p* < 0.0001) (Table 2).

Additionally, *APOE* ε4, a risk gene for various dementia-related diseases, along with P/LP Variants, constitutes the genetic foundation of dementia. We therefore tested whether *APOE* ε4 carriage inversely affected P/LP Variant detection. *APOE* ε4 carriers had a significantly lower P/LP Variant detection rate than non-carriers (5.1% vs. 14.1%; *p* = 0.00035).

Due to limited repeated follow-up neuropsychological data, MMSE and MoCA progression rates showed no significant association with P/LP Variant detection. Therefore, we excluded cognitive progression measures from the final model to maintain sample size.

To enhance clinical applicability, we binarized RelNum at >2 versus ≤2 and compared its performance and calibration with the continuous RelNum model (Supplementary Table S6). The dichotomized RelNum achieved the lowest AIC of 250.87 and remained a significant predictor (*p* = 0.0034), indicating superior model fit. We next tested whether including disease category in a multivariable logistic regression with AAO, RelNum, parental disease status, EarlyFH, and *APOE* ε4 would improve prediction. Disease category was nonsignificant (*p* > 0.05), increased AIC, and reduced AUC versus the model with only AAO, RelNum, parental disease status, EarlyFH, and *APOE* ε4 (Supplementary Table S5). These results support the simpler model’s applicability across dementia subtypes.

Finally, we selected AAO, EarlyFH, RelNum, parental disease status, and *APOE* ε4 as the predictors in a multivariate logistic regression. For clinical convenience, we further transformed AAO into binarized variables at cutpoints of 50, 55, 60, 65, and 70 years. In addition, we explored three-bin models with cutpoints at 50–60, 55–65, and 65–85, comparing each to the continuous AAO model.

### 3.1. Prediction of Model in the Derivation Cohort

The multivariable regression results are detailed in Supplementary Tables S4 and S5. A clinical prediction model for dementia was developed to identify variables associated with P/LP Variant detection. Regression coefficients, standard errors, z-values, and *p*-values appear in Supplementary Table S2, and odds ratios with 95% confidence intervals for each predictor are listed in Supplementary Table S3.

**Figure 3:**
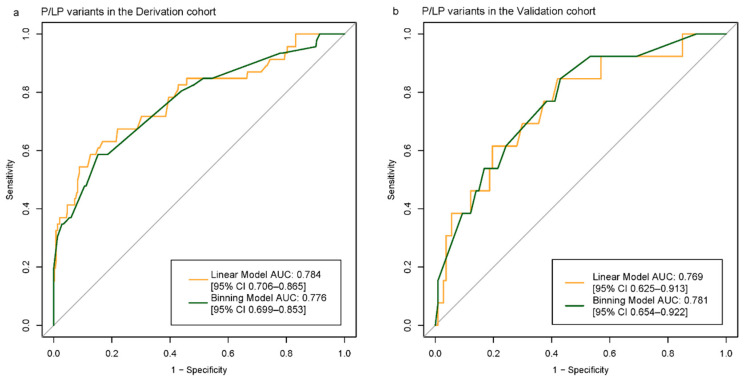
ROC curves for P/LP Variant detection among participants with a positive family history of dementia in the derivation (Panel (**a**)) and validation (Panel (**b**)) cohorts. Both continuous-AAO (orange) and AAO ≥ 55 binning (green) models include EarlyFH, AAO, RelNum, parental disease status, and *APOE* ε4 carriage.

We fitted a multivariable logistic regression model in the derivation cohort using EarlyFH, AAO, RelNum, parental disease status, and *APOE* ε4 carriage. In the continuous-AAO model, the AAO coefficient was –0.0396 (*p* = 0.0027; OR 0.961, 95% CI 0.927–0.994), with AUC 0.784 (95% CI 0.706–0.865) and AIC 246.51. Binarizing AAO at 55 years produced AUC 0.776 (95% CI 0.699–0.853) and AIC 249.56, with AAO ≥55 significantly associated with P/LP Variant detection (*p* = 0.0098) (Figure 3a). EarlyFH, RelNum >2, and parental disease status were positively associated with P/LP detection, while *APOE* ε4 carriage was inversely associated (Supplementary Tables S2 and S3). Compared with the Goldman score model, our final model achieved superior discrimination and calibration, although calibration plots indicated slight overestimation at high predicted probabilities. Patient distributions by predicted risk category are shown in Supplementary Table S4, and binning model parameters in Supplementary Equation (S1).

### 3.2. Model Performance in Validation Cohort

**Figure 4:**
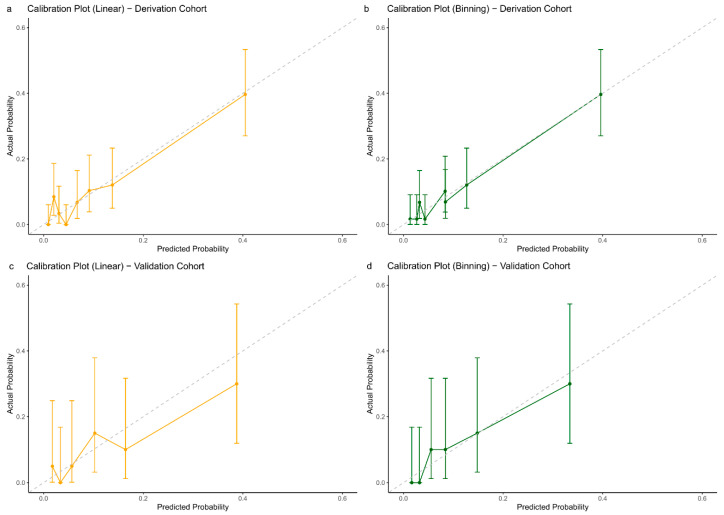
Calibration plots for P/LP Variant detection among participants with a family history of dementia. The vertical axis represents observed incidence (actual probability) and the horizontal axis represents predicted probability; (**a**) Calibration plot of linear model in derivation cohort. (**b**) Calibration plot of binning model in derivation cohort. (**c**) Calibration plot of linear model in validation cohort. (**d**) Calibration plot of binning model in validation cohort.

Both models performed well on the validation set. The binning model achieved an AUC of 0.769 (95% CI, 0.625–0.913). In contrast, treating AAO as a continuous variable resulted in an AUC of 0.781 (95% CI, 0.654–0.922) (Figure 3b). All models were well calibrated, with Hosmer–Lemeshow test *p*-values greater than 0.05 and ICI values less than 0.2 (Figure 4a–d).

### 3.3. Subgroup Performance by Inheritance Mode

We next assessed performance separately in autosomal-dominant (AD) and autosomal-recessive (AR) carriers versus non-carriers. In the AD subgroup, the model achieved an AUC of 0.84 (95% CI 0.76–0.91) and demonstrated good calibration (Hosmer–Lemeshow χ^2^ = 3.00, *p* = 0.56). At a probability threshold of 0.1, sensitivity was 73.7% and specificity 79.7%, while at 0.2 sensitivity decreased to 60.5% and specificity increased to 89.0%. In the AR subgroup, discriminative performance was lower (AUC = 0.60, 95% CI 0.41–0.79) with poor calibration (Hosmer–Lemeshow χ^2^ = 30.23, *p* < 0.001); sensitivity was 30.0% at a 0.1 threshold and 20.0% at 0.2, with specificity of 79.7% and 89.0%, respectively. ROC and calibration plots for both subgroups are provided in Supplementary Figure S2.

### 3.4. Model Performance Compared to the Previously Published Goldman Score

In the derivation cohort, the AUC was 0.667 [95% CI, 0.588–0.745] for the Goldman score model and 0.718 [95% CI, 0.640–0.796] for the AAO + Goldman model. In the validation cohort, the AUC was 0.695 [95% CI, 0.556–0.834] for the Goldman score model and 0.688 [95% CI, 0.528–0.849] for the AAO + Goldman model. At the 11.0% probability threshold for P/LP detection, the clinical-based P/LP model improved event reclassification by 32.9% [95% CI, 30.6–59.5%] compared to the Goldman model [17] and by 12.9% [95% CI, 3.2–74.3%] compared to the Goldman + AAO model; for nonevents, it improved by 4.3% [95% CI, −3.6–12.1%] compared to the Goldman model and by 14.4% [95% CI, 5.3–30.1%] compared to the Goldman + AAO model.

### 3.5. Application of Discriminant Models in Clinical Genetic Testing Decisions

Our model stratifies individuals by P/LP Variant risk after dementia diagnosis. Selection of a decision threshold should balance clinical yield and cost. At a 10% cutoff, 26.9% of individuals are classified as high risk, yielding a positive predictive value (PPV) of 18.9% (95% CI 9.4–32.0%) and a negative predictive value (NPV) of 95.5% (95% CI 87.5–99.1%); increasing the threshold to 20% reduces the high-risk group to 12.0%, with PPV of 25.0% (95% CI 9.7–46.7%) and NPV of 92.7% (95% CI 85.6–97.0%). A nomogram for individualized risk estimation is provided in Supplementary Figure S1.

### 3.6. Decision Curve Analysis

**Figure 5:**
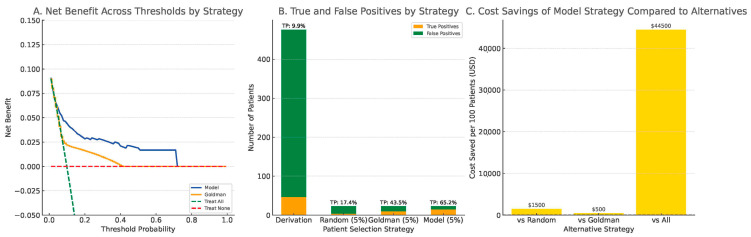
Clinical utility and cost-saving assessment of the predictive model. (**A**) decision curve analysis comparing net benefit of the model versus the Goldman score, treat-all, and treat-none strategies. (**B**) true positives (orange) and false positives (green) under four selection strategies, with TP proportions labeled. (**C**) estimated cost savings per 100 patients assuming US$500 per WES test.

Decision curve analysis (DCA) in the derivation cohort showed that the clinical model provided greater net benefit across threshold probabilities between 0.05 and 0.60 compared to the Goldman score, the treat-all, and treat-none strategies (Figure 5A).

To simulate clinical decision-making under testing resource constraints, we compared four strategies, assuming that only 5% of patients could be selected for whole-exome sequencing (WES): random selection, top 5% by Goldman score, and top 5% by our model. The model-based strategy identified 65.2% of all true Pathogenic/Likely Pathogenic (P/LP) carriers, compared to 43.5% by the Goldman-based strategy and only 17.4% by random selection (Figure 5B).

Cost simulation, using a conservative estimate of US$500 per WES test based on published clinical sequencing costs [30], demonstrated that the model-based strategy could reduce unnecessary testing expenditures by up to US$44,500 per 100 patients compared to universal testing. It also showed superior economic efficiency relative to both random selection and Goldman-based prioritization (Figure 5C). These findings underscore the model’s practical value in optimizing resource allocation and improving diagnostic efficiency in real-world clinical settings.

## 4. Discussion

Using data from a PUMCH dementia clinic collected between January 2014 and June 2022, we developed the first clinical information-based model to identify patients at high risk of carrying P/LP Variants associated with dementia via WES. The model was subsequently validated using data from the same clinic collected between July 2022 and August 2024. The final model incorporates the following variables: EarlyFH (family history of early onset case ≥ 1 vs. =0), AAO (age at onset ≤ 55 vs. >55), RelNum (number of affected family members < 3 vs. ≥3), parent (parental disease status: present vs. absent), and *APOE* (*APOE* ε4 carrier status: present vs. absent), demonstrating good predictive discrimination and calibration.

Previous literature has extensively explored the relationship between age, family history, and genetics, with family history often analyzed qualitatively through scoring systems such as the Goldman score. Later studies incorporated age as a categorical variable to discriminate high-risk P/LP Variant carriers among patients with dementia [30]. However, a comprehensive predictive model with demonstrated discrimination and calibration has yet to be developed. Meanwhile, we identified limitations in the clinical application of the Goldman score, including gaps in its applicability and challenges in score interpretation.

Given the high costs and long sequencing times associated with WES, this study aims to utilize easily accessible clinical indicators to predict the probability of detecting P/LP Variants and guide genetic testing decisions. To facilitate rapid clinical application, we converted the variables into categorical variables whenever possible. For variable processing, both a linear model and a binning model were developed to enhance the model’s interpretability, and the two models were compared in terms of model performance and calibration. The binning model showed performance and calibration comparable to the linear model.

Although the study cohort in this research is a single-center cohort (the PUMCH dementia cohort), the derivation cohort comprised outpatient data from January 2014 to June 2022, while the validation set comprised outpatient data from July 2022 to August 2024, ensuring temporal validation of the model. The participants in this cohort come from 33 provinces, municipalities, and autonomous regions across China, providing good representation across different institutions and regions.

AAO has long been considered a key factor associated with carrying P/LP Variants. We initially applied restricted cubic splines to evaluate the relationship between AAO and P/LP Variant carriage, but found that the nonlinear component did not significantly improve model fit, indicating a good linear relationship. For better clinical applicability, we then proceeded to categorized AAO. Previous studies have often classified dementia onset into early-onset and late-onset categories based on the age of 65, with some suggesting that the threshold for identifying high-risk P/LP Variant carriers should be <60 years. Based on clinical experience and previous literature, we explored AAO cutoffs at 50, 55, 60, 65, and 70 in both two-bin and three-bin models (Supplementary Table S7), then selected the optimal model (AAO cutoff at 55) according to performance, calibration, and AIC.

We also found that the presence of *APOE* ε4 in patients with dementia and a positive family history was inversely associated with the likelihood of carrying P/LP Variants. This suggests distinct disease patterns for *APOE* ε4 carriers and carriers of P/LP Variants. Therefore, we consider *APOE* ε4 as an independent factor negatively correlated with P/LP Variant carriage.

We also found that EarlyFH, RelNum, and pedigree information were associated with P/LP Variant detection. Due to the challenges in fully investigating family pedigrees, we used parental disease status as a substitute, which proved to be relevant. EarlyFH, based on patient and family recall, often reflect the age when symptoms became more apparent, so we extended the age threshold to 65 years. For RelNum, univariate analysis showed no significant difference in P/LP Variant detection rates for fewer than three affected relatives. In multivariate analysis, using a cutoff of two provided the best model performance and calibration; therefore, we defined multiple affected relatives as at least three.

With increasing attention to dementia-related diseases and the widespread adoption of WES, more patients with dementia and their families are expressing concerns about genetic predisposition. We developed and validated a model that provides individualized estimates of the probability of carrying P/LP Variants. The model flags high-risk patients for WES, which can confirm diagnosis, inform prognosis, and guide family counseling. It also spares low-risk patients unnecessary testing. Applying a probability threshold of 0.2 yielded a negative predictive value of 92.7% (95% CI, 85.6–97.0%) and specificity of 90.0% (95% CI, 87.2–92.8%), supporting its use to guide genetic testing decisions and alleviate concerns about genetic predisposition. Moreover, decision curve analysis confirmed that our model delivers superior net clinical benefit across relevant thresholds, reinforcing its practical value in prioritizing patients for WES.

### Limitations and Future Directions

This study has three main limitations. First, although overall discrimination and calibration were good, sensitivity was lower than specificity and performance was reduced in autosomal-recessive carriers. We recommend a decision threshold of 0.2, which was selected based on clinical context, the observed risk distribution, and DCA. Further validation in larger AR cohorts is required. Second, as a single-center study at a tertiary specialty hospital in China with limited longitudinal neuropsychological data (e.g., ADL decline), generalizability to primary care, community settings, and other populations remains to be demonstrated; multi-center studies and richer follow-up data are needed. Third, variant classification relied on a December 2024 snapshot of ClinVar and OMIM; because genotype–phenotype databases are updated frequently and new pathogenic variants continue to emerge, carrier frequency estimates should be periodically refreshed.

Future work should expand AR sample sizes, integrate longitudinal cognitive assessments, validate the model across diverse healthcare settings, and routinely update variant annotations to maintain accuracy.

## 5. Conclusions

We developed and validated a model using AAO, EarlyFH, RelNum, parental disease status, and *APOE* ε4 carrier status to predict P/LP Variant carriage in patients with dementia and a positive family history. The model demonstrated strong discrimination, good calibration, and clinical net benefit on DCA. A 0.2 probability threshold balances predictive value and resource use, identifying high-risk individuals for genetic testing while sparing low-risk individuals unnecessary testing. Subgroup analysis showed weaker performance in AR carriers, indicating a need for larger cohorts. Periodic updating of variant annotations as genotype–phenotype databases (e.g., ClinVar, HGMD) evolve is recommended to maintain accuracy. Further multi-center validation and integration of longitudinal cognitive data will enhance generalizability. In conclusion, this tool provides a practical framework for guiding genetic testing and hereditary counseling.

## Figures and Tables

**Table 1:** Clinical characteristics of patients with cognitive impairment in this study.

Clinical Features	FH *N* = 601	Derivation *N* = 476	Validation *N* = 125	*p*-Value
AAO, years	65 (57–72)	66 (58–73)	58 (52–67)	<0.001
<55	115 (19.13%)	76 (15.97%)	39 (31.20%)	<0.003
55–64	178 (29.62%)	140 (29.41%)	38 (30.40%)	0.826
65–84	296 (49.25%)	250 (52.52%)	46 (36.80%)	0.002
>85	6 (1.00%)	5 (1.05%)	1 (0.80%)	>0.99
Gender (M, %)	248 (41.26%)	196 (41.18%)	52 (41.60%)	>0.99
Disease duration, years	3 (2–5)	4 (2–5.5)	3 (2–5)	0.965
Education attainment, years	11 (7–13.13)	11 (7–14)	9 (7–12)	0.095
*APOE*				
*APOE* ε4/ε4	50 (8.32%)	40 (8.40%)	10 (8.00%)	>0.99
*APOE* ε4/-	254 (42.26%)	204 (42.86%)	50 (40.00%)	0.611
Family history				
Goldman score	3.5 (3.5–3.5)	3.5 (3.5–3.5)	3 (3–4)	0.032
EarlyFH Parent disease status RelNum	107 (17.8%)	74 (15.55%)	33 (26.4%)	<0.001
	458 (76.21%)	364 (76.47%)	94 (75.2%)	0.814
	1 (1–2)	1 (1–2)	1 (1–2)	0.613
Cognition				
MMSE	17 (10–23)	18 (11–24)	14 (6.75–21)	<0.001
MoCA	16 (12–20)	16 (12–21)	14 (10–18.25)	0.026
ADL	34 (27–44)	34 (26–44)	35.5 (29–45)	0.052
P/LP	62 (10.32%)	49 (10.29%)	13 (10.40%)	>0.99

Data are median (IQR) for continuous variables (AAO, disease duration, education, Goldman score, MMSE, MoCA, ADL) and *n* (%) for categorical variables (AAO categories, gender, *APOE* ε4 genotypes, FH, EarlyFH, parental disease status, RelNum, P/LP variant carriers); between-group comparisons used Mann–Whitney U test for continuous and Fisher’s exact test for categorical variables.

**Table 2:** Distribution of P/LP Variant carriers by clinical and demographic subgroups.

Participants	Group	Subgroup	Total Cases	P/LP (Count, %)	Fisher’s Exact Test *p*-Value
FH+ (*n* = 601)	RelNum	>2	60	18 (30.00%)	<0.0001
		≤2	541	44 (8.13%)	
	AAO	≤55	134	27 (20.15%)	<0.0001
		>55	467	35 (7.49%)	
	*APOE* ε4	0	347	49 (14.12%)	0.00035
		1	254	13 (5.12%)	
	Parental Disease Status	Dementia-Affected	458	55 (12.01%)	0.017
		Dementia-Unaffected	143	7 (4.90%)	
	EarlyFH	With Early-Onset Cases	122	30 (24.59%)	<0.0001
		Without Early-Onset Cases	479	32 (6.68%)	
	Gender	Male	248	29 (11.69%)	0.414
		Female	353	33 (9.35%)	
	Goldman Score	1	35	15 (42.86%)	<0.0001
		2	22	3 (13.64%)	
		3	90	12 (13.33%)	
		3.5	454	32 (7.05%)	
	Disease category	AD	409	31 (7.58%)	0.0072
		FTD	45	5 (11.11%)	
		VaD	63	11 (17.46%)	
		Other	84	15 (17.86%)	

All values are *n* (%); between-group comparisons used Fisher’s exact test; AAO = age at onset; EarlyFH = presence of any family member with dementia onset < 65 years; RelNum = number of affected relatives (excluding the index patient); Parental disease status = at least one parent affected by dementia; *APOE* ε4 includes ε4/ε4 and ε4/– genotypes; P/LP Variant = Pathogenic/Likely Pathogenic Variant; FH+ = patients with a family history of dementia; disease category abbreviations: AD = Alzheimer’s disease; FTD = frontotemporal dementia; VaD = vascular dementia; Other = other dementia subtypes.

## Data Availability

The data supporting the findings of this study are available from the corresponding author upon reasonable request. The data are not publicly available due to privacy and ethical restrictions.

## Supplementary Materials

The following supporting information can be downloaded at: https://www.mdpi.com/article/10.3390/biomedicines13051235/s1, Equation (S1). Regression equation used to binning model. Table S1: Summary of clinical features of dementia patients in this study based on clinical diagnosis. Table S2: Coefficients and Significance of Clinical Variables in the Binning Model. Table S3: Odds Ratios and 95% Confidence Intervals. Table S4: Distribution of patients and events within pre-specified predicted risk categories in the derivation cohort. Table S5: Comparison of Simple Model vs. Disease Diagnosis Model. Table S6: Comparison of Continuous vs. Binning RelNum Models. Table S7: Summary of Logistic Regression Models with Different AAO Transformations. Table S8: Baseline Clinical and Genetic Characteristics of 62 Pathogenic/Likely Pathogenic Variant Carriers Identified by Whole-Exome Sequencing. Figure S1: Nomogram for Predicting P/LP Variant Carriage in Dementia Patients with a Positive Family History. Figure S2: ROC and calibration plots for P/LP variant detection in autosomal dominant (AD) and autosomal recessive (AR) subgroups.
