# Supplementary material for: Predictive Accuracy of a Clinical Model for Carriage of Pathogenic/Likely Pathogenic Variants in Patients with Dementia and a Positive Family History at PUMCH
Source: Biomedicines. 2025 May 19;13(5):1235. doi: 10.3390/biomedicines13051235 (PMC12108604; doi:10.3390/biomedicines13051235)
Supplement: Supplementary file 1 [file biomedicines-13-01235-s001.zip › Supplement Equlator.pdf]

Supplement Equation S1

$$\text{logit}(P) = -2.8819 + 0.9609\times\text{EarlyFH} + 0.9365\times\text{AAO} + 1.1996\times\text{RelNum} + 1.5504\times\text{parent} - 1.0159\times \textit{APOE}$$

Where:

- EarlyFH is a binary variable for family history early onset case ≥ 1 (1) or $=0$ (0)
- AAO is a binary variable for age onset ≤ 55 (1) or >55 (0)
- RelNum is a binary variable for the number of affected family members < 3 (0) or ≥ 3 (1)
- parent is a binary variable for parental disease status (1 for present, 0 for absent)
- *APOE* is a binary variable for *APOE* ε4 carrier status (1 for present, 0 for absent)
