# Supplementary material for: Predictive Accuracy of a Clinical Model for Carriage of Pathogenic/Likely Pathogenic Variants in Patients with Dementia and a Positive Family History at PUMCH
Source: Biomedicines. 2025 May 19;13(5):1235. doi: 10.3390/biomedicines13051235 (PMC12108604; doi:10.3390/biomedicines13051235)
Supplement: Supplementary file 1 [file biomedicines-13-01235-s001.zip › Supplement Figure.pdf]

Supplement Figure S1: Nomogram for Predicting P/LP Variant Carriage in Dementia Patients with a Positive Family History.

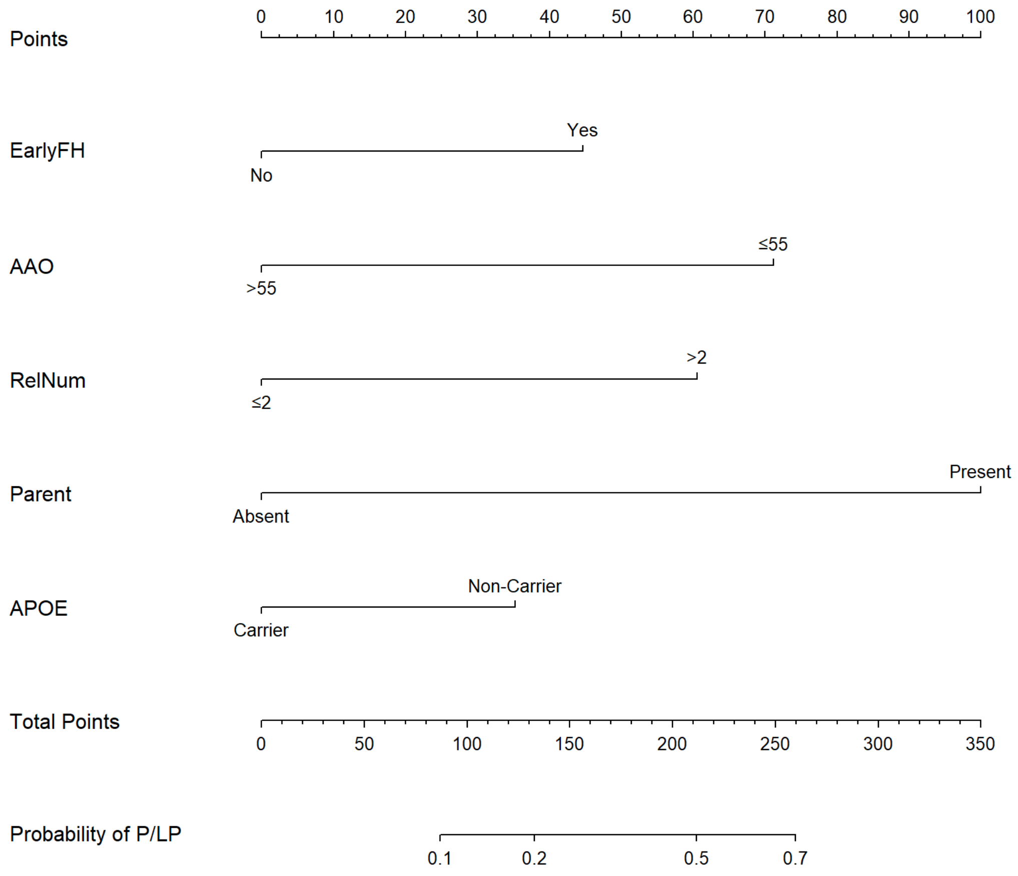

This nomogram was constructed using a binning logistic regression model that incorporates early family history (EarlyFH), age at onset (AAO, dichotomized at 55 years), number of affected relatives (RelNum; >2 vs. ≤2), parental disease status, and APOE ε4 carrier status. Each variable is assigned a score proportional to its regression coefficient, and the cumulative score is converted into a predicted probability of detecting pathogenic/likely pathogenic variants. This tool facilitates individualized risk stratification to guide genetic testing decisions in clinical practice.

Supplement Figure S2: ROC and calibration plots for P/LP variant detection in autosomal dominant (AD) and autosomal recessive (AR) subgroups.

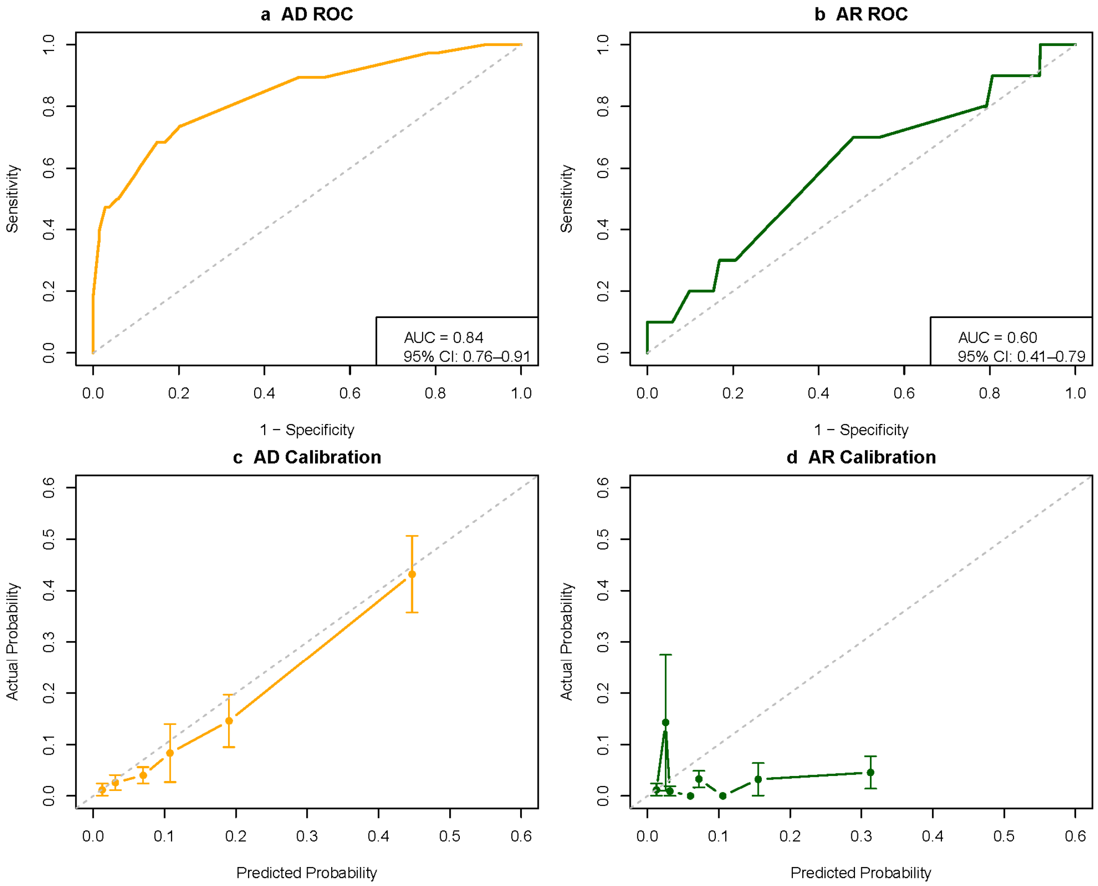

Panels (a–b) show ROC curves (sensitivity vs 1 − specificity) for the AD (a) and AR (b) subgroups; panels (c–d) show calibration plots (observed event rate vs predicted probability) for the AD (c) and AR (d) subgroups.
