# Supplementary material for: Predictive Accuracy of a Clinical Model for Carriage of Pathogenic/Likely Pathogenic Variants in Patients with Dementia and a Positive Family History at PUMCH
Source: Biomedicines. 2025 May 19;13(5):1235. doi: 10.3390/biomedicines13051235 (PMC12108604; doi:10.3390/biomedicines13051235)
Supplement: Supplementary file 1 [file biomedicines-13-01235-s001.zip › Supplement Table.pdf]

Supplement Table S1: Summary of clinical features of dementia patients in this study based on clinical diagnosis.				
Clinical features	AD<br>N = 409	FTD<br>N = 45	VaD<br>N = 63	Other dementia<br>N = 84
AAO, years	65 (57–74)	60 (54–66)	66 (58.5–71.5)	62 (53.5–69)
<55	66 (16.1%)	12 (26.7%)	13 (20.6%)	24 (28.6%)
55-64	119 (29.1%)	19 (42.2%)	15 (23.8%)	25 (29.8%)
65-84	214 (52.3%)	14 (31.1%)	34 (54.0%)	34 (40.5%)
>85	5 (1.22%)	0 (0%)	1 (1.59%)	0 (0%)
Gender (M, %)	158 (38.6%)	19 (42.2%)	31 (49.21%)	40 (47.62%)
Disease duration, years	3 (2–5)	4 (2–6)	3 (1.5–5)	2 (1–4.75)
Education attainment, years	11(8-13)	10 (7–14)	10 (7–12)	11(8-14)
APOE				
APOE ε4/ε4	44 (10.8%)	0 (0%)	4 (6.35%)	2(2.38%)
APOE ε4/-	203 (49.6%)	14 (31.1%)	18 (28.57%)	19(22.62%)
Family history				
Goldman score	3.26±0.33	3.24±0.36	3.13±0.56	3.08±0.69
EarlyFH	68(16.6%)	7(15.6%)	16(25.4%)	16(19.05%)
Parent disease status	305(74.6%)	36(80%)	52(82.54%)	65(77.38%)
RelNum	1(1-2)	1(1-2)	1(1-2)	1(1-2)
Cognition				
MMSE	14.5(9-20)	13.5(7-20)	19(14.5-24)	19(12.5-25)
MoCA	14(10-18)	14(10-18)	16(12-20)	16(11.5-20)
ADL	37(30-45)	38(29-47)	36(28.5-44.5)	40(30-50.5)
P/LP	31 (7.58%)	5 (11.11%)	11 (17.46%)	15(17.86%)

The age at onset, the age at diagnosis, disease duration, educational attainment, MMSE/MoCA/ADL scores are all shown as median(q1-q3). Goldman score are shown as mean±standard deviation. Gender, family history, *APOE* genotypes P/LP are all shown as numbers and proportions (%). AD Alzheimer's disease, FTD frontotemporal dementia,VaD vascular dementia, ONDD other neurodegenerative diseases , nonAD non-AD dementias. MMSE Mini-Mental State Examination. MoCA Montreal Cognitive Assessment. ADL Activities of Daily Living. P/LP Pathogenic/likely Pathogenic variants.

Supplement Table S2: Coefficients and Significance of Clinical Variables in the Binning Model				
Variable	Estimate	Std. Error	z-value	Pr(>|z|)
Intercept	−2.8819	0.6773	-4.255	<0.0001(***)
EarlyFH	0.9609	0.3708	2.592	0.0096(**)
AAO	0.9365	0.3626	2.583	0.0098(**)
RelNum	1.1996	0.4160	2.884	0.0039(**)
Parent disease status	1.5504	0.6387	2.427	0.015(*)
APOE	-1.0159	0.3541	-2.869	0.0041(**)

The table presents the results of the multivariate regression analysis. The variables included are: Early case (family history early onset case ≥ 1 vs =0, AAO (age of onset ≤ 55 vs >55), number (number of affected family members < 3 vs ≥3), parent (parental disease status: present vs absent), and *APOE* (*APOE* ε4 carrier status: present vs absent).

Significance codes: *** p < 0.001; ** p < 0.01; * p < 0.05.

The null deviance was 306.83, and the residual deviance was 238.56, with an AIC value of 250.40.

Supplement Table S3: Odds Ratios and 95% Confidence Intervals

Variable	Odds Ratio	2.5% CI	97.5% CI
Intercept	0.06	0.02	0.21
EarlyFH	2.61	1.26	5.41
AAO	2.56	1.25	5.26
RelNum	3.32	1.47	7.50
Parent disease status	4.72	1.35	16.47
APOE	0.36	0.18	0.73

The table presents the odds ratios (OR) and 95% confidence intervals (CI) for the clinical variables included in the multivariate regression model. An OR greater than 1 indicates an increased likelihood of the outcome, while an OR less than 1 suggests a decreased likelihood. Family history early case, number of affected family members ⩾ 3, parent disease status and AAO<55 were associated with higher odds of the outcome, whereas *APOE* ε4 carrier status was associated with lower odds.

Supplement Table S4: Distribution of patients and events within pre-specified predicted risk categories in the derivation cohort

Predicted risk category	< 1%	1-4.9%	5-9.9%	10-19.9%	>20%
Binning Model					
No. of patients	34	189	118	71	57
No. of events	0	7	9	8	23
Continuous Model					
No. of patients	0	206	149	50	71
No. of events		8	10	5	25
Goldman Model					
No. of patients	0	0	370	61	45
No. of events	0	0	23	8	16
Goldman + AAO Model					
No. of patients	0	274	40	123	39
No. of events	0	14	5	15	13

The Goldman Model is a multivariate regression model based on the Goldman Score. The Goldman + AAO model, based on Koriath 2020, improves upon the Goldman classification by incorporating age at onset (AAO), with the AAO threshold set at 60 years.

Supplement Table S5: Comparison of Simple Model vs. Disease Diagnosis Model

	Derivation AUC	Validation AUC	H-L test	AIC	Variable Significance
Model^{S}	0.786	0.757	0.79	250.87	All variables are significant (p < 0.05)
Model^{D}	0.772	0.752	0.91	254.98	The additional variable, diagnosis catagory, is not significant (p > 0.05)

Model^{s} includes five variables (AAO, *APOE*, RelNum, Parental disease status, and earlyFH), whereas Model^{d} includes these five variables plus an additional variable, diagnosis_catagory. The diagnosis_catagory variable is based on clinical and biological diagnoses, coded as follows: 1 represents AD, 2 represents FTD, 3 represents other dementias, and 4 represents VaD. The HL-Test (Hosmer-Lemeshow Test) is used to assess calibration in the validation cohort.

Supplement Table S6: Comparison of Continuous vs. Binning RelNum Models					
Model	RelNum Significance (p)	Training AUC	Testing AUC	H-L Test	AIC
Model[C]	0.011	0.7843	0.7696	0.40	251.90
Model[B]					
Model[1]	0.045	0.7854	0.7888	0.44	254.39
Model[2]	0.0034	0.7863	0.7574	0.40	250.87
Model[3]	0.29	0.7819	0.8088	0.33	257.23
Model[4]	0.43	0.7831	0.8017	0.57	257.65

The Model[C] treats RelNum as a continuous predictor, whereas the Model[B] convert RelNum into a binary variable based on thresholds of >1, >2, >3, and >4, respectively.

Supplement Table S7: Summary of Logistic Regression Models with Different AAO Transformations						
Model	AAO Transformation	Derivation AUC	Validation AUC	H-L Test	AIC	AAO p-value
Model 1: Linear AAO	Continuous	0.7843	0.7696	0.3983	246.51	0.00270
Model 2: AAO Binarized (threshold > 50)	AAO > 50 vs. ≤ 50	0.7699	0.7665	0.7140	254.04	0.16071
Model 2: AAO Binarized (threshold > 55)	AAO > 55 vs. ≤ 55	0.7761	0.7812	0.49768	249.56	0.00980
Model 2: AAO Binarized (threshold > 60)	AAO > 60 vs. ≤ 60	0.7811	0.7763	0.66713	249.85	0.01391
Model 2: AAO Binarized (threshold > 65)	AAO > 65 vs. ≤ 65	0.7787	0.7785	0.88584	251.17	0.03526
Model 2: AAO Binarized (threshold > 70)	AAO > 70 vs. ≤ 70	0.7654	0.7669	0.58974	255.50	0.54276
Model 3: AAO Three-Bin (50–60)	cutpoints: 50, 60	0.7809	0.7714	NA	251.77	Medium: 0.776; High: 0.05445
Model 3: AAO Three-Bin (55–65)	cutpoints: 55, 65	0.7835	0.7834	0.05343	250.35	Medium: 0.093; High: 0.00638
Model 3: AAO Three-Bin (65–85)	cutpoints: 65, 85	0.7793	0.7785	0.88777	252.96	Medium: 0.037; High: 0.990

Model 1 used AAO as a continuous variable in a linear model, Model 2 used a two-category binning of AAO, and Model 3 used a three-category binning. The Hosmer-Lemeshow (H-L) test evaluated calibration in the validation cohorts.
